# *Pseudomonas fluorescens *alters epithelial permeability and translocates across Caco-2/TC7 intestinal cells

**DOI:** 10.1186/1757-4749-2-16

**Published:** 2010-11-27

**Authors:** Amar Madi, Pascal Svinareff, Nicole Orange, Marc GJ Feuilloley, Nathalie Connil

**Affiliations:** 1 LMDF-SME, Laboratory of Cold Microbiology-Signals and Microenvironment, UPRES EA 4312, 55 rue Saint Germain, 27000 Evreux, France; 2 BIOGALENYS, 9 Rue de Pacy, 27930 Miserey, France

## Abstract

**Background:**

*Pseudomonas fluorescens *has long been considered as a psychrotrophic microorganism. Recently, we have shown that clinical strains of *P. fluorescens *(biovar 1) are able to adapt at a growth temperature of 37°C or above and induce a specific inflammatory response. Interestingly, a highly specific antigen of *P. fluorescens*, I2, is detected in the serum of patients with Crohn's disease but the possible role of this bacterium in the disease has not yet been explored. In the present study, we examined the ability of a psychrotrophic and a clinical strain of *P. fluorescens *to modulate the permeability of a Caco-2/TC7 intestinal epithelial model, reorganize the actin cytoskeleton, invade the target cells and translocate across the epithelium. The behaviour of these two strains was compared to that of the well known opportunistic pathogen *P. aeruginosa *PAO1.

**Results:**

Both strains of *P. fluorescens *were found to decrease the transepithelial resistance (TER) of Caco-2/TC7 differentiated monolayers. This was associated with an increase in paracellular permeability and F-actin microfilaments rearrangements. Moreover, the invasion and translocation tests demonstrated that the two strains used in this study can invade and translocate across the differentiated Caco-2/TC7 cell monolayers.

**Conclusions:**

The present work shows for the first time, that *P. fluorescens *is able to alter the intestinal epithelial barrier function by disorganizing the F-actin microfilament network. Moreover, we reveal that independently of their origins, the two *P. fluorescens *strains can translocate across differentiated Caco-2/TC7 cell monolayers by using the transcellular pathway. These findings could, at least in part, explain the presence of the *P. fluorescens *specific I2 antigen in the serum of patients with Crohn's disease.

## Background

*Pseudomonas fluorescens *has long been considered as a psychrotrophic microorganism, unable to grow at temperatures above 32°C. Recently, we have shown that clinical strains of *P. fluorescens *(biovar 1) have been able to adapt at a growth temperature of 37°C or above [1]. This event appears relatively frequently and can lead to strains of increased virulence, in the range of the well known opportunistic pathogen *Pseudomonas aeruginosa*. Some of these clinical strains of *P. fluorescens *have a high hemolytic activity [2] presumably associated with type III or VI secretion systems and were shown to induce cytotoxic and proinflammatory responses on epithelial intestinal cells [3]. These observations and the incidence of declared nosocomial infections due to this germ [4] have changed the vision of the potential risk associated with *P. fluorescens*. This gram-negative bacterium is widespread in the environment where it is even more frequent than *P. aeruginosa *and has a remarkable fitness potential [5]. *P. fluorescens *is found in many ecological niches including soil and water but also in refrigerated food products where its psychrotrophic character gives it the possibility to grow in the relative absence of competitors [6].

*P. fluorescens *can also be found as a low level commensal of the human digestive tract [7]. Interestingly, a highly specific antigen of *P. fluorescens*, designated as I2, was detected in the serum of 54% of the patients suffering from ileal Crohn's disease (CD) [8] and a correlation between the severity of the pathology and the level of the circulating I2 antigen has been demonstrated [9]. A relation between exposure to psychrotrophic germs and CD is suggested by the parallel emergence and rise of the pathology in western countries and the widespread use of refrigerated food [10]. Although CD, as other chronic inflammatory bowel diseases, is typically a multifactorial pathology, these data underline the interest of better understanding of the possible interactions between *P. fluorescens *and the intestinal epithelium.

Bacterial pathogens can cause exacerbations or relapse of ulcerative colitis and CD. A healthy intestinal epithelium acts as a barrier between foreign antigens and the underlying lymphoid tissue. Many pathogens have developed strategies to circumvent the barrier function of an intact epithelium. These include inflammatory induction, physical disruption of the barrier and crossing the epithelial cell monolayer. The barrier function is maintained by tight junctions, which are dynamic cell-cell adhesions that form a continuous seal around the cells and control the permeability of solutes and fluids diffusing through the paracellular pathway [11]. Tight junctions are formed by transmembrane proteins, such as claudins and occludin, and cytosolic proteins recruited to the apicolateral membrane, including zonula occludens proteins, cingulin and 7H6 [12,13]. Cytoskeletal components are anchored to tight junction structures through the cytosolic proteins, forming a perijunctional acto-myosin ring which gives the junction architectural support. These components of the cytoskeleton may also be involved in shuttling signalling molecules to and from the junctions [14,15]. Consequently, tight junctions can be disrupted by rearrangement and contraction of the perijunctional acto-myosin ring and this could lead to variations in transepithelial resistance (TER). Alteration of the epithelial barrier function by pathogens can be associated with translocation of the microorganisms across the monolayer. Such mechanisms are used by many enteropathogens, like *Salmonella*, *Yersinia*, *Shigella *or *Listeria*, to disseminate within the host. Three translocation mechanisms are relevant to the intestinal epithelium: *(i) *exploitation of M cells, specialized intestinal epithelial cells which transport luminal antigens across the epithelial barrier to underlying lymphoid tissue; [16] transcellular translocation or trancytosis, in which pathogens invade epithelial cells, migrate across the cytoplasm and egress from the opposite surface; and *(iii) *paracellular translocation or paracytosis, in which bacteria travel extracellulary through tight junctions between adjacent epithelial cells. Translocation across intestinal epithelial cells barriers via all these three mechanisms has been reported for *Campylobacter jejuni *which in turn promotes the translocation of other commensal bacteria [17,18].

We have recently shown that *P. fluorescens *can adhere to intestinal epithelial cells, has cytotoxic activity and induces a specific inflammatory response [3], but the ability of this bacterium to alter the intestinal epithelial barrier function or to translocate across this epithelium has not yet been studied until now. In the present study, we examined the ability of a psychrotrophic and a clinical strain of *P. fluorescens *to modulate the permeability of a Caco-2/TC7 intestinal epithelial model, reorganize the actin cytoskeleton, translocate across the epithelium and invade the target cells. The behaviour of these two strains was compared to that of the well known opportunistic pathogen *P. aeruginosa *PAO1.

## Methods

### Bacterial strains

*P. fluorescens *MFN1032 (biovar I) was collected in a hospital of Haute-Normandie (France). It was characterized by polyphasic identification, 16 S RNA sequencing and siderotyping [1]. *P. fluorescens *MF37 is a spontaneous rifampicin-resistant mutant of the strain MF0 (biovar V), originally identified in crude milk [19]. *P. aeruginosa *PAO1 was obtained from an international collection. All the strains were cultivated in ordinary nutrient broth (Merk, Darmstadt, Germany), at 28°C for the two strains of *P. fluorescens *or 37°C for *P. aeruginosa *PAO1.

### Cell line and culture

The Caco-2/TC7 clone established from the parental human enterocytes-like Caco-2 cell line [20] was used between passages 25 and 35. Cells were routinely grown in Dulbecco's Modified Eagle's Medium (DMEM, invitrogen) supplemented with 15% heat-inactived fetal calf serum, 2 mM of L-glutamine, 100 U/ml each of penicillin and streptomycin and 1% non essential amino acids. For experimental assays, the cells were seeded at a density of approximately 10^5 ^cells/cm^2 ^in 24-well tissue culture plates or on inserts (6.4 mm diameter, 3 μm pore size, Falcon) which allow epithelial differentiation between apical and basolateral compartments. The cells were cultured at 37°C in 5% CO_2_-95% air atmosphere and the medium was changed daily. Caco-2/TC7 cells grown in 24-well tissue culture plates were incubated to early confluence (undifferentiated cells). Caco-2/TC7 cells grown on inserts were used at 21 days post-confluence (fully differentiated cells).

### Cell infection

Bacteria in early stationary phase (grown overnight) were harvested by centrifugation (5000 g, 5 min, 20°C) and resuspended at a density of 10^8 ^colony forming unit (CFU)/ml in cell culture medium without serum and antibiotics. Caco-2/TC7 grown on 24-well culture plates (undifferentiated cells) or inserts (fully differentiated cells) were washed twice with fresh cell culture medium and the bacterial suspensions were applied to the surface or to the apical compartments at a concentration of 10^8 ^CFU/cm^2^. Infected cells were then incubated at 37°C in 5% CO_2_-95% air during 24 h for permeability, 3 or 6 h for translocation and 4 h for invasion assays. Each assay was conducted in triplicate in independent experiments (successive passages of Caco-2/TC7 cells).

### Transepithelial resistance measurements

The transepithelial resistance (TER) of differentiated Caco-2/TC7 monolayers was studied during 24 h, using the Millicell Electrical Resistance System (Millipore Corp, Bedford, MA). TER values are expressed as percentages of the pre-infection level of the TER (baseline) measured for each individual cell monolayer in the inserts.

### Cytotoxicity assay

At the end of infection, the supernatants from Caco-2/TC7 monolayers were collected and the concentration of lactate dehydrogenase (LDH), a cytoplasmic enzyme released upon cell death, was determined using an enzymatic assay (Cytotox 96 Promega, Charbonnieres, France) as previously described [3].

### Quantification of paracellular flux

Paracellular permeability was quantified using the fluorescein isothiocyanate dextran tracer 4 kDa (FD-4). One hour before the end of infection, 1 mg/ml of FD-4 was added to the apical side of differentiated Caco-2/TC7 monolayers and the amount of FD-4 translocated to the basolateral medium was then measured by fluorimetry using a TD700 fluorometer (Turner Designs) (485 nm excitation and 528 nm emission wavelengths). The results are expressed as a percentage of apical dextran that crossed the insert membrane per cm^2 ^and per hour as described by Lynch *et al *[21].

### Actin visualisation

At the end of infection, apical F-actin cytoskeleton was visualized. Briefly, differentiated Caco-2/TC7 monolayers were washed with phosphate-buffered saline (PBS), fixed for 10 min in 3.7% paraformaldehyde and permeabilized for 5 min with 0.1% Triton X100 at room temperature. The cells were incubated with 1% bovine serum albumine in PBS for 10 min. F-actin was stained by incubation with Alexa-488 phalloïdine (1 U/insert) for 45 min at room temperature. Following three washes in PBS, cell monolayers were excised from the filter supports, mounted on slides using Fluoromount/Plus mounting medium (Diagnostic BioSystems) and examined using a confocal laser scanning microscope (Zeiss, LSM710).

### Bacterial translocation

After 3 and 6 h of bacterial infection, aliquots of 100 μl of the basolateral compartment were collected and the number of bacteria that crossed the epithelial monolayers was determined by serial dilution and plating onto nutrient agar. *P. fluorescens *and *P. aeruginosa *are aerobic bacteria which require free access to oxygen for a rapid multiplication. Indeed, control studies showed that in our experimental conditions, the growth of *P. fluorescens *and *P. aeruginosa *in the basolateral compartment of the inserts was negligible. This observation allows to consider that the bacterial population measured in the basolateral compartment is actually corresponding to bacteria that crossed the epithelial barrier.

### Bacterial invasion assay

After 4 h of infection, Caco-2/TC7 monolayers grown in 24-well culture plates or inserts were washed with PBS. Adherent bacteria were killed by incubation for 1 h with 300 μg/ml gentamycin, an antibiotic that does not cross the cytoplasmic membrane of eukaryotic cells and then only kills bacteria not internalized in cells. Caco-2/TC7 monolayers were washed 3 times with PBS to remove the antibiotic and dead bacteria and were lysed by incubation for 15 min with 0.1% Triton X100 to release the intracellular bacteria. The lysates were then plated onto nutrient agar to determine the number of internalized bacteria.

### Statistical analysis

All experiments were conducted independently at least three times. The results are expressed as means ± standard error of the mean [22] and statistical significance were performed by ANOVA with Bonferroni post hoc test.

## Results

### *P. fluorescens *can alter the epithelial barrier function

**Figure 1:**
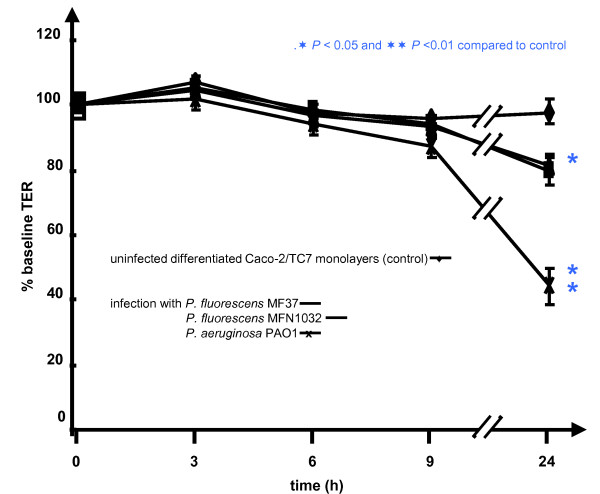
***Pseudomonas fluorescens *MF37, *P. fluorescens *MFN1032 and *P. aeruginosa *PAO1 decrease the transepithelial resistance of Caco-2/TC7 cell monolayers**: TER measurements after infection with *P. fluorescens *MF37, *P. fluorescens *MFN1032 and *P. aeruginosa *PAO1 compared to uninfected differentiated Caco-2/TC7 monolayers (control). The data are expressed as percentage of the initial TER measured across the monolayer at the onset of the experiment. Results are the means values ± standard errors of the means (SEM) for at least three independent experiments.

The effect of *P. fluorescens *MF37, *P. fluorescens *MFN1032 and *P. aeruginosa *PAO1 infection on epithelial permeability was evaluated by measuring the TER across differentiated Caco-2/TC7 monolayers (Figure 1). TER values were measured at the onset of the experiment and at times 3, 6, 9 and 24 h. Up to 9 h after the beginning of the experiment, the TER values of the monolayers exposed to *P. fluorescens *MF37, *P. fluorescens *MFN1032 and *P. aeruginosa *PAO1 remained unchanged. After 24 h of infection, the TER of the monolayers exposed to the two strains of *P. fluorescens *were significantly decreased (-20.3 ± 3.9% for MF37 and -18.3 ± 3.1% for MFN1032). *P. aeruginosa *led to a deeper decrease of the TER value (55.8 ± 5.3%). The fall in TER can not be attributed to damages provoked by acidification of the medium since the pH of the medium remained constant over the studies.

### The decrease of TER is associated with an increase in paracellular permeability

**Figure 2:**
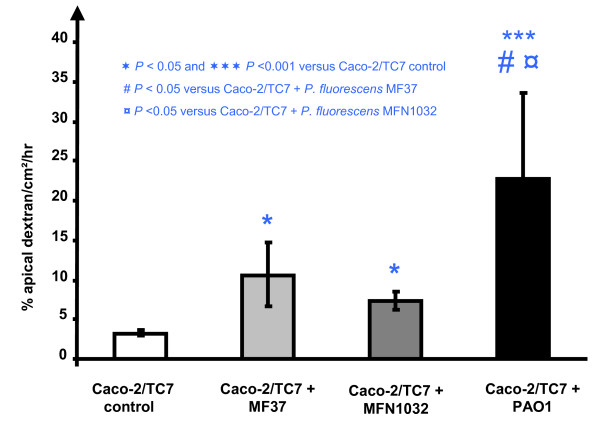
**The TER decrease is accompanied by an increase in paracellular permeability**. Apical-to-basolateral FD-4 flux in response to infection with *P. fluorescens *MF37, *P. fluorescens *MFN1032 and *P. aeruginosa *PAO1. The data are the percentages of apical FD-4 crossing the insert membrane per cm^2 ^per hour and are the means values ± SEM of at least three independent experiments.

The observed decrease in TER can have three causes: *(i) *an increase of paracellular permeability, [16] cell lysis and epithelium disorganization or *(iii) *variations of ion fluxes across an intact monolayer. To distinguish between these possibilities, cell lysis was estimated by an assay of LDH released upon cell death after 24 h of infection and the paracellular permeability was quantified by measuring the apical to basolateral flux using 4 kDa dextran tracer (FD-4). Only *P. aeruginosa *PAO1 had a marginal cytotoxic effect on differentiated Caco-2/TC7 cell monolayers after 24 h of incubation (data not shown). In contrast, the three tested bacteria provoked a significant increase in the paracellular permeability after 24 h of incubation (Figure 2). The paracellular permeability of control differentiated Caco-2/TC7 monolayers was low (3.2 ± 0.3% of apical FD4 crossing the epithelial barrier/cm^2^/h). *P. aeruginosa *PAO1 caused the greatest increase of paracellular permeability with 22.8 ± 10.8% of apical FD-4 crossing the barrier/cm^2^/h. The effect of *P. fluorescens *MF37 and *P. fluorescens *MFN1032 was less pronounced but reached 10.6 ± 4.1% and 7.4 ± 1.2% FD-4 crossing/cm^2^/h, respectively.

### *P. fluorescens *induces apical F-actin filaments reorganization

**Figure 3:**
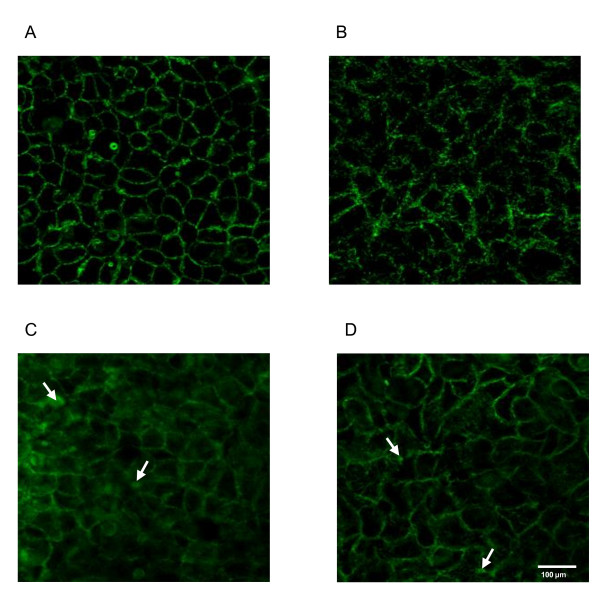
***Pseudomonas fluorescens *MF37, *P. fluorescens *MFN1032 and *P. aeruginosa *PAO1 cause disruption of the apical F-actin cytoskeleton**. Fluorescent F-actin staining showing organized F-actin filaments lining the apical tight-junction system of control differentiated Caco-2/TC7 cell monolayer (A). In cell monolayers infected with *P. fluorescens *MF37 (B), *P. fluorescens *MFN1032 (C) and *P. aeruginosa *PAO1 (D) the sub-membrane microfilament network is loose. Clusters of amorphous actin can be observed, particularly after infection with *P. fluorescens *MFN1032 and *P. aeruginosa *PAO1 (arrows).

Because F-actin filaments in the apical poles of epithelial cells are intimately linked to and regulate tight junction functions, we looked at the effect of *P. fluorescens *MF37, *P. fluorescens *MFN1032 and *P. aeruginosa *PAO1 on the organization of the sub-membrane microfilament network. As shown in Figure 3, incubation with bacteria induced a dramatic reorganization of apical epithelial F-actin. The staining pattern of untreated Caco-2/TC7 showed a continuous fine meshwork of microfilaments lining the cell border. In cells exposed for 24 h to the three bacterial strains, the normal organization of microfilaments was replaced by a diffused and loose network of F-actin fibers. Moreover, these changes were accompanied by the formation of dense clusters of apparently amorphous F-actin, particularly after treatment with the clinical strain *P. fluorescens *MFN1032 and with *P. aeruginosa *PAO1.

### *P. fluorescens *translocates across Caco-2/TC7 monolayers

**Figure 4:**
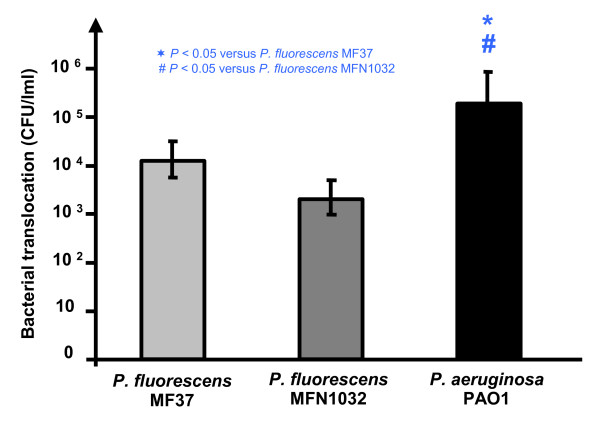
***Pseudomonas fluorescens *MF37, *P. fluorescens *MFN1032 and *P. aeruginosa *PAO1 can translocate across differentiated Caco-2/TC7 cell monolayers**. Bacteria inoculated onto the apical surface of differentiated Caco-2/TC7 cell monolayers and having translocated to the basolateral compartment were quantified after 6 h of infection. Results are expressed as the mean number of translocating bacteria counted after plating (CFU/ml) ± SEM in at least three independent experiments.

After apical infection of differentiated Caco-2/TC7 monolayers, bacterial strains were quantified in the basolateral medium at different times. After three hours of infection, bacteria were not detectable in the basolateral compartment. However, after 6 h of infection, 1.5 ± 2.2 × 10^4^, 2.9 ± 2.5 × 10^3 ^and 7.2 ± 2.2 × 10^5 ^CFU/ml were detected with *P. fluorescens *MF37, *P. fluorescens *MFN1032 and *P. aeruginosa *PAO1, respectively (Figure 4). These values of bacterial translocation remain marginal compared to the size of the initial inoculum (10^8 ^CFU). Nevertheless, if we consider that these results indicate that 1/1000 to 1/10,000 of *Pseudomonas *can cross the epithelium within 6 h, these values may be highly significant in a situation of chronic exposure to these bacteria.

### *P. fluorescens *can translocate without disrupting Caco-2/TC7 cell monolayer integrity

Bacterial translocation through the monolayer can result either from trans- or paracellular passage of the bacteria or more simply from gaps formed in the monolayer following the death of some epithelial cells. The TER was evaluated at different times and, as previously shown in Figure 1, this value remained identical for all tested bacteria between the onset of the experiment and 9 h of incubation. This absence of variation of TER indicates that the monolayer integrity was maintained at least 9 h after the infection. These results are in agreement with measures taken using FD-4 between 0 and 9 h of incubation with bacteria that showed no significant variations of paracellular permeability during these short incubation periods (data not shown).

### *P. fluorescens *can be invasive in Caco-2/TC7 cells

**Figure 5:**
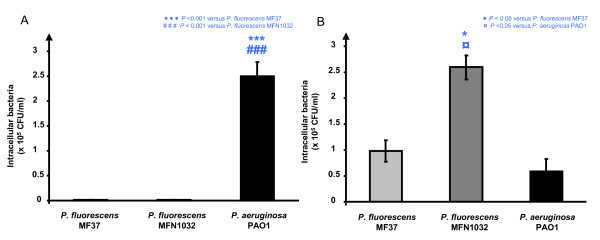
***Pseudomonas fluorescens *MF37, *P. fluorescens *MFN1032 and *P. aeruginosa *PAO1 can be invasive in Caco-2/TC7 cells**. Undifferentiated (A) or differentiated (B) Caco-2/TC7 cells were infected for 4 h and intracellular bacteria were quantified by plating after gentamicin destruction of extracellular germs. Results are expressed as the mean number of bacteria (CFU/ml) ± SEM recovered in the intracellular compartment in at least three independent experiments.

Since the differentiated Caco-2/TC7 monolayer remained intact and the paracellular permeability was unchanged at times when bacteria were actually recovered in the basolateral compartment, these results suggested that *Pseudomonas *are able to invade epithelial cells, migrate across the cytoplasm and egress at the level of the basal membrane. This possible transcellular translocation of *Pseudomonas *was investigated using the gentamicin exclusion test. The behaviour of bacteria was different in undifferentiated and differentiated cells. In undifferentiated Caco-2/TC7 cells, only *P. aeruginosa *PAO1 was invasive (2.5 × 10^5 ^CFU/ml) whereas no *P. fluorescens *MF37, or *P. fluorescens *MFN1032 were detected in the intracellular compartment (Figure 5A). The situation was different in differentiated Caco-2/TC7 monolayers where the three bacterial strains had clearly an invasive behaviour with 0.9 ± 0.2 × 10^5^, 2.6 ± 0.2 × 10^5 ^and 0.6 ± 0.3 × 10^5 ^CFU/ml detected in the intracellular compartment for *P. fluorescens *MF37, *P. fluorescens *MFN1032 and *P. aeruginosa *PAO1, respectively (Figure 5B). It is interesting to note that in the differentiated monolayer, the two strains of *P. fluorescens *were more invasive that *P. aeruginosa *PAO1, and the level of the clinical strain of *P. fluorescens *MFN1032 was more than 4 times that of the *P. aeruginosa *strain

## Discussion

Precise regulation of intestinal epithelial tight junctions is crucial to maintain the barrier function between the luminal compartment and the interior medium. Numerous mucosal pathogens can modulate tight junctions and translocate across the epithelial barrier leading to established disease states. In this regard, our pioneer works [23,24] and the recent studies demonstrating that *P. fluorescens *can clearly act as an opportunistic pathogen [3,25] underscore the need to further characterize the virulence potential of this microorganism. In the present work, we studied the translocation and invasion potential of *P. fluorescens *on differentiated Caco-2/TC7 epithelial intestinal cell monolayers and the effect of the bacteria on paracellular permeability and tight junctions associated actin cytoskeleton. The behaviour of these bacteria was compared to that of the opportunistic pathogen *P. aeruginosa *PAO1.

We demonstrate that, independently of their maximal growth temperature, the two strains of *P. fluorescens *MF37 and MFN1032, respectively from environmental and clinical origin, can lead to a time-dependent decrease of the epithelial barrier function. This effect starts after 9 h of contact between the bacteria and the epithelial cells but is more pronounced at 24 h post-infection. This decrease of TER is lower than that induced by *P. aeruginosa *PAO1. This result is logical since it has been demonstrated that the *P. aeruginosa *quorum sensing factor N-(3 oxodecanoyl)-L-homoserine lactone can disrupt the epithelial barrier integrity of Caco-2 cells [26] whereas *P. fluorescens *is not known to produce N-acyl homoserine lactones. The quorum sensing factors expressed by this species remain to be identified. Compared to some other bacteria such as *Escherichia coli *O157 which are known to cause a rapid decrease in TER [27], the decrease of TER observed after treatment with the two *P. fluorescens *strains presently studied was slow, suggesting that this effect on TER is mediated by synthesis of active molecules either by epithelial cells and/or bacteria. Indeed, *P. fluorescens*, and particularly a strain such as MFN1032, is known to produce exoenzymes but also cyclo-lipopeptides [28]. On the other hand, it is now obvious that many eukaryotic communication factors, including peptides locally produced by the intestinal epithelium, can enhance the virulence of *Pseudomonas *[29]. A decrease of TER can result either from an increase in paracellular permeability, local cell lysis in the monolayer or change in ion flux across the intact monolayer. To distinguish between these possibilities, LDH released by lysed cells was quantified and fluorescent markers of paracellular flux were used. We have previously shown that *P. fluorescens *MF37 and *P. fluorescens *MFN1032 can exert a cytotoxic effect on confluent undifferentiated epithelial intestinal cells [3]. Surprisingly, on fully differentiated Caco-2/TC7 cells cultivated on insert, no significant cytotoxicity was observed, even after 24 h of infection with these two strains. This indicates that the increase of TER is not due to direct toxic effects and cell lysis. Only infection with *P. aeruginosa *PAO1 provoked a modest lysis of fully differentiated Caco-2/TC7 cells explaining the higher effect of this strain on the TER values. It has been shown that, as the epithelial barrier differentiates and becomes highly polarized, it is more resistant to *P. aeruginosa *infection [30]. This could explain the rather low cytotoxicity of the three *Pseudomonas *species or strains presently studied in comparison with our previous results obtained on undifferentiated cells [3].

The integrity of the epithelial cell monolayer is maintained by intercellular junctional complexes composed of tight junctions, adherens junctions and desmosomes. The tight junctions are the most apical intercellular junctions. They form a continuous belt-like structure at the luminal end of intercellular space that regulates the paracellular flux [31]. Some bacterial pathogens can manipulate the apical-junctional complex and one of the principal strategies used by these microorganisms is to trigger actin cytoskeleton contraction [32]. In the present study, the confocal laser scanning microscope examination of apical F-actin revealed that *P. fluorescens *MF37, *P. fluorescens *MFN1032 and *P. aeruginosa *PAO1 induced a profound reorganization of the enterocyte actin cytoskeleton. This effect is very similar to that reported in cells exposed to *Vibrio parahaemolyticus *[21] or enteropathogenic *E. coli *[33]. These observations are in agreement with the results obtained by measurement of TER and FD-4 flux across the cell monolayer.

Our recent observations indicate that *P. fluorescens *induces the secretion of the proinflammatory cytokine interleukin (IL)-8 in epithelial intestinal cells [3]. IL-6, IL-1β and TNF-α secretion are also induced in human cultured A549 pulmonary cells by *P. fluorescens *[25]. Proinflammatory cytokines released in the gut can increase paracellular permeability by modulating tight junction protein expression and phosphorylation [34]. Moreover, modest inflammatory and metabolic stress are known to promote the translocation and transcytosis of commensal bacteria such as *E. coli *C25 across gut enterocytes [35]. Consequently, we investigated the ability of *P. fluorescens *MF37, *P. fluorescens *MFN1032 and *P. aeruginosa *PAO1 to translocate across the Caco-2/TC7 cell monolayers. We found that although the translocation is limited compared to the initial inoculum, these bacteria were observed to readily translocate across Caco-2/TC7 monolayers after 6 h of infection. Most importantly, TER measurements and FD-4 flux indicate that at this time, the cell monolayer integrity was maintained in the presence of the translocating bacteria. Numerous mucosal bacteria, such as *Salmonella typhymurium*, translocate across the epithelial barrier and this translocation is associated with a complete loss of TER after 6 h [36]. Pathogen bacteria of the *Pseudomonas *genus are generally considered as extracellular microorganisms. However, these bacteria have been shown to have an invasive behaviour in specific eukaryotic cells such as corneal epithelial cells for *P. aeruginosa *[37] and cortical neurons for *P. fluorescens *[38]. Our results suggest that the translocation of *Pseudomonas*, through the cell monolayers, occurs after invasion of the differentiated cells. It is known that *S. typhymurium *and *E. coli *M12 are more invasive in immature undifferentiated than in differentiated Caco-2 cells [39]. In contrast, in our study the gentamicin exclusion test demonstrates that while *P. aeruginosa *PAO1 invade both polarized and non-polarized Caco-2/TC7 cells, *P. fluorescens *only invades the differentiated cells. As expected for a clinical strain, *P. fluorescens *MFN1032 had a higher invasive activity than its psychrotrophic and environmental counterpart *P. fluorescens *MF37. However, despite this higher invasion potential, the translocation level of *P. fluorescens *MFN1032 was similar to that of MF37. These observations can be explained by the fact that polarized epithelial cells are characterized by the presence of membrane proteins distributed to apical or basolateral surfaces, and these proteins may be absent or poorly expressed in undifferentiated epithelial cells. Then, bacteria may preferentially interact with these proteins, exhibiting affinity for either apical or basolateral surfaces, or for differentiated or undifferentiated epithelial cells [40].

## Conclusion

Our results reveal that the psychrotrophic strain *P. fluorescens *MF37 and the clinical strain *P. fluorescens *MFN1032 have very similar effects on Caco-2/TC7 cells and can contribute to a decrease in the barrier effect of the intestinal epithelium. As these bacteria have a low cytotoxic activity, in these cells, they apparently disorganize intercellular junctions by acting on the F-actin microfilament network. Moreover, we have shown that independently of its origins and physiological variations, *P. fluorescens *can translocate across differentiated Caco-2/TC7 cell monolayers by using the transcellular pathway. These findings could, at least in part, explain the presence of the *P. fluorescens *specific I2 antigen in the serum of patient with CD. Further studies of the effects of this bacterium on the expression and distribution of tight junction proteins of intestinal epithelial cells, may help to understand the molecular mechanism used by this bacterium to modulate the intestinal epithelial barrier function.

## Competing interests

The authors declare that they have no competing interests.

## Authors' contributions

AM carried out most experiments and analyzed most of the data. NC wrote the manuscript and participated in the design of the study. PS and NO helped to draft the manuscript. MF participated in the design of the study and writing of the manuscript. All authors read and approved the final manuscript.

## Aknowledgments

This study was supported by grants from the Conseil Général de l'Eure, the Communauté d'Agglomération d'Evreux and Europe. LMDF-SME is a member and supported by the world's leading center *Cosmetic Valley*. AM is a recipient of a doctoral fellowship from the Région Haute-Normandie and BioGalenys.

The authors wish to thank Christine Farmer for English reviewing of the manuscript.
